# Prevention of Hydrogen Damage Using MoS_2_ Coating on Iron Surface

**DOI:** 10.3390/nano9030382

**Published:** 2019-03-06

**Authors:** Xiaolong Li, Li Chen, Hongmei Liu, Changmin Shi, Dongchao Wang, Zhishan Mi, Lijie Qiao

**Affiliations:** 1 Beijing Advanced Innovation Center for Materials Genome Engineering, Corrosion and Protection Center, University of Science and Technology Beijing, Beijing 100083, China; lixiaolong@lyu.edu.cn (X.L.); mizhishan@163.com (Z.M.); 2 Institute of Condensed Matter Physics, Linyi University, Linyi 276000, China; liuhongmei@lyu.edu.cn (H.L.); shichangmin@lyu.edu.cn (C.S.); wangdongchao@lyu.edu.cn (D.W.)

**Keywords:** hydrogen adsorption, MoS_2_, protective barrier, strain, work function

## Abstract

The prevention of hydrogen penetration into steels can effectively protect steels from hydrogen damage. In this study, we investigated the effect of a monolayer MoS_2_ coating on hydrogen prevention using first-principles calculations. We found that monolayer MoS_2_ can effectively inhibit the dissociative adsorption of hydrogen molecules on an Fe(111) surface by forming a S–H bond. MoS_2_ coating acts as an energy barrier, interrupting hydrogen penetration. Furthermore, compared with the H-adsorbed Fe(111) film, the work function of the MoS_2_-coated film significantly increases under both equilibrium and strained conditions, indicating that the strained Fe(111) film with the MoS_2_ coating also becomes more corrosion resistant. The results reveal that MoS_2_ film is an effective coating to prevent hydrogen damage in steels.

## 1. Introduction

Hydrogen damage is a severe problem since hydrogen degrades the mechanical properties of steels [1]. For example, hydrogen can induce low yield and fracture stresses in steels. Hydrogen atoms in structural materials usually come from the reduction of hydrogen ions or dissociative adsorptions of some H-containing gases, such as H_2_, H_2_O, and H_2_S. The prevention of hydrogen penetration into steels can effectively protect steels from hydrogen damage, which is typically achieved by applying a thin protective coating of TiN/TiC [2], SiC [3], an aluminum or a chromium rich layer [4,5,6], some alloy coatings [7], and conductive polymers [8] all of which show resistance to hydrogen or hydrogen isotopic permeability. It has been reported that AlN coatings [9] and Er_2_O_3_ coatings on 316L stainless steel [10] and MoS_2_/Ni_80_Cr_20_ films on pure iron [11] can act as protective barriers of metals against hydrogen or hydrogen isotope permeation. The graphene coating was found to decrease the hydrogen embrittlement susceptibility of the metal substrate, as the hydrogen content in graphene-coated copper was greatly reduced after hydrogen charging [12,13]. It is thought that less hydrogen is introduced to the bulk material by using these surface coatings.

Among various protection coatings, MoS_2_ possesses excellent physical and chemical properties that are suitable for preventing hydrogen permeation into metals. MoS_2_ not only has a 1185 °C melting point but is also chemically stable at an ambient atmosphere up to 315 °C. A good mechanical strength has also been reported for monolayer MoS_2_, which is a flexible and strong material with a high Young’s modulus comparable to steels [14]. Furthermore, monolayer MoS_2_ is a semiconductor with a direct band gap of ∼1.8 eV [15], and the low electrical conductivity and nearly insulating channel of MoS_2_ are also advantageous for hydrogen permeation barriers. For monolayer MoS_2_, the Mo atoms and S atoms combine with each other by covalent bonds. When coated on metals, the MoS_2_ layer usually exhibits a good adhesive performance, because S atoms can firmly bind to a metal surface [16]. The stable metal–S interface leads to a high diffusion barrier for hydrogen atoms to overcome in MoS_2_ coatings than that in metals. It is thought to protect the underlying metal substrate from corrosion and oxidation. Recently, MoS_2_ coatings of a few micrometers thick on pure iron substrates have been fabricated by magnetron sputtering [11]. However, the current understanding of MoS_2_ on steels as a protective barrier against hydrogen damage is still limited, which has motivated the present study.

More specifically, steels usually experience a moderate strain in the service environment due to external mechanical loads and residual stresses, which can affect the mechanical and electronic properties of steels [17,18]. Experimental studies have suggested that stress corrosion cracking and hydrogen embrittlement are dominating damages for steels. Thus, strain and hydrogen are two important factors to alter the physical properties of steels. The protective properties of MoS_2_ coating on iron films have also been investigated with applied stress and hydrogen.

Here we explore monolayer MoS_2_ as a promising coating for the protection of steels against hydrogen damage using first-principles calculations. To simulate the coating effects of MoS_2_ on steels, we chose pure iron film as a substrate, instead of steels with various additional elements, to provide a basic understanding. We found that the MoS_2_ monolayer can stably bind to the Fe(111) surface and effectively inhibit the dissociative adsorption and permeation of hydrogen. MoS_2_ interrupts hydrogen penetration by the formation of S–H bonds. In addition, compared with the H-adsorbed Fe(111) film, the work function of the MoS_2_-coated film significantly increases under both equilibrium and strained conditions. The present results suggest the feasibility of MoS_2_ coating as a protective barrier against hydrogen damage.

## 2. Calculation Methods

All calculations reported in this work were performed in the framework of the spin-polarized density functional theory (DFT), as implemented in the Vienna ab initio simulation package (VASP, 5.4.1, Universität Wien, Wien, Austria) [19]. The electron–ion interaction was described using the projector augmented wave (PAW) method [20]. The exchange correlation between electrons was treated with generalized gradient approximation (GGA) in the Perdew–Burke–Ernzerhof (PBE) form [21]. Using a 15 × 15 × 15 *k*-point mesh for the primitive cell, we obtained for body-centered cubic (bcc) Fe a lattice constant of 2.835 Å and a local magnetic moment of 2.20 μ_B_ per Fe atom, which agree very well with the experimental values of 2.866 Å [22] and the theoretical results performed with different *k*-point meshes [23,24,25,26].

**Figure 1:**
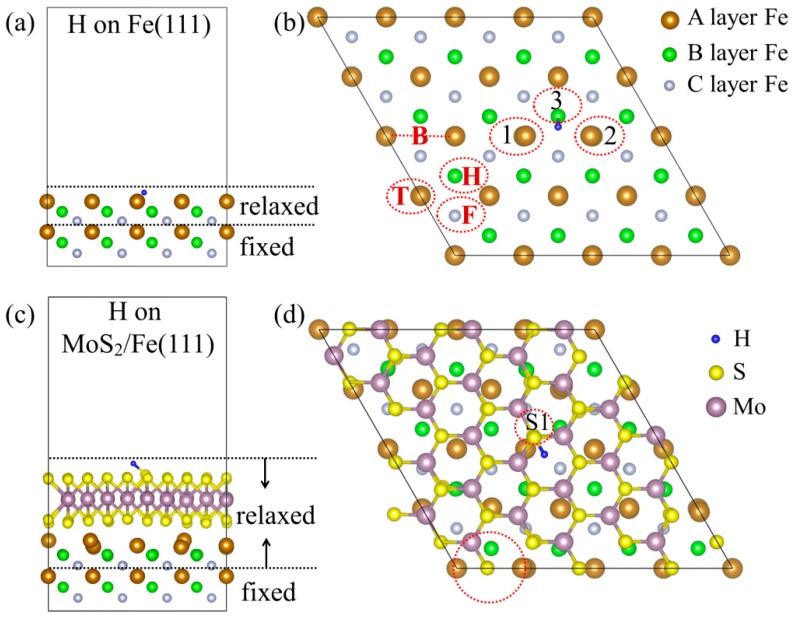
(**a**) Side and (**b**) top views of H-adsorbed Fe(111) film with six layers. The brown, green, and silver balls represent Fe atoms in the A, B, and C layer, respectively. The possible adsorption sites of H are marked in (b), on the top site of surface Fe (T), the bridge site of two surface Fe atoms (B), the hcp (hexagonal close packed) hollow site (H), and the fcc (face-centered cubic) hollow site (F). The numbers 1, 2, and 3 in (b) present the Fe1, Fe2, and Fe3 atoms that surround the H atom. (**c**) Side and (**d**) top views of H-adsorbed MoS_2_/Fe(111) film. For the MoS_2_ and Fe(111) interfaces, one S atom (noted in the red dotted circle in (d)) located at the bridge site of the surface Fe atoms is the most energetically stable configuration.

For the study of H adsorption on the iron surface, we used a 4 × 4 surface cell of Fe(111) surface with six layers of Fe atoms, as shown in Figure 1a,b. A plane-wave energy cutoff of 400 eV with a *k*-point sampling of 3 × 3 × 1 in the Brillouin zone (BZ) was employed for the 4 × 4 surface cell. A vacuum region of 15 Å was introduced to eliminate the electronic interactions between the periodic images. The convergence criterion for the energy was set to 10^−5^ eV. For structural optimization, the bottom three Fe layers were fixed at their bulk positions and other atoms were fully relaxed until the force on each atom was lower than 0.01 eV/Å. In the case of asymmetric models, owing to the different electronegativity of two neighboring surfaces, a dipolar interaction appears in the z direction and affects the work function. To eliminate such interactions between the periodic replicas, dipole corrections were employed in the z direction for asymmetric slabs by using the method proposed by Neugebauer and Scheffler [27].

## 3. Results

When the H atom approaches the Fe(111) surface, we found that H chemically adsorbs on Fe atoms. Figure 1a,b displays the geometric structure of the film with one H atom adsorption on the surface. The H atom prefers to adsorb at the bridge site of the two top layer Fe atoms, such as Fe1 and Fe2 presented in Figure 1b. Table 1 lists the adsorption energy, *E*_ads_, which is defined as

(1) $$E_{\mathrm{ads}}=E\left(H/\mathrm{film}\right)-E(\mathrm{film})-\frac{1}{2}E(H_{2})$$

where *E*(H/film) and *E*(film) are the energy of the Fe(111) film (or MoS_2_/Fe film) with or without H adsorption. The energy of H is referenced to the H_2_ molecule and hence reflects a dissociative adsorption energy. The adsorption energy for H adsorbing at the bridge site was −0.55 eV, suggesting that the H_2_ molecule can easily dissociate on the Fe(111) surface. Previous theoretical calculations have predicted that H adsorption energy on Fe(110) and Fe(100) surfaces is −0.71 eV and −0.38 eV [23], respectively. The H atom can form a strong chemical bond with the Fe3 atom with a distance of 1.636 Å, which is in good agreement with the literature results that report the closest H–Fe distance as 1.66 Å for H on Fe(110) and 1.68 Å for H on Fe(100) [23].

**Figure 2:**
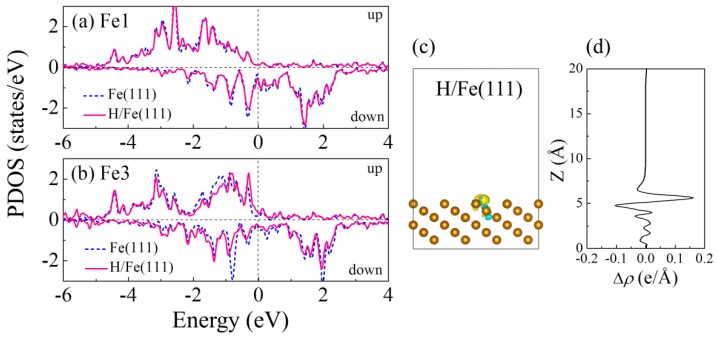
Projected density of states (PDOS) onto (**a**) Fe1 and (**b**) Fe3 atoms in the clean Fe(111) film and the H-adsorbed Fe(111) film, respectively. (**c**) Charge transfer density between a H atom and Fe(111). The yellow and blue colors represent the electron accumulation and depletion, respectively. The charge density isosurface was set to 0.003 e Å^−3^. (**d**) Interfacial charge transfer between a H atom and the Fe(111) film as a function of the z coordinate perpendicular to the surface.

The chemisorbed H atom binds to the Fe(111) surface with a strong orbital overlap, which significantly affects the density of states (DOS) of the nearby Fe atoms, as shown in Figure 2. The H atom initially locates at the bridge site of the Fe1 and Fe2 atoms, and then binds to the Fe3 atom after geometric optimization. From Figure 2a, we observe that the projected density of states (PDOS) on Fe1 near the Fermi level slightly decreases after H adsorption, while the closest Fe3 exhibits an evident PDOS reduction as shown in Figure 2b—in particular the down spin. The decrease of Fe PDOS reflects the reduction of the charge density of Fe atoms, as the H atom withdraws 0.355 e from the Fe(111) surface after adsorption, as listed in Table 1. To further illustrate the detailed nature of the charge transfer, we show in Figure 2c the charge difference between the H-adsorbed Fe(111) system and the sum of the isolated Fe(111) and the H atom, which is defined as

(2) $$\Delta \rho =\rho_{H/\mathrm{Fe}}-(\rho_{\mathrm{Fe}}+\rho_{H})$$

where $\rho_{H/\mathrm{Fe}}$, $\rho_{\mathrm{Fe}}$, and $\rho_{H}$ are the charge density of the H/Fe(111) film, the Fe(111) film, and the H atom at the same lattice constant with frozen atom positions. The yellow regions represent the charge accumulation and the blue regions represent the depletion of electrons. To have a quantitative picture, we plot in Figure 2d the plane-averaged electron density difference along the perpendicular direction (z) to the Fe(111) surface. As seen in Figure 2c,d, the Fe(111) film acts as a donor and the H acts as an acceptor with electrons transferring from the Fe to the H due to the difference in electronegativity.

The above results show that hydrogen molecules can easily dissociate on the clean Fe surface and permeate into the bulk, as reported in literature [23]. The accumulation of H in bulk can generate hydrogen bubbles which are harmful for steels. In order to inhibit hydrogen permeation, a monolayer MoS_2_ is coated on the Fe(111) surface. For the model, a $3\sqrt{3}\times 3\sqrt{3}$ MoS_2_ supercell was constructed in a hexagonal geometry on six layers of 4 × 4 Fe(111) film, as shown in Figure 1c,d. We have considered the MoS_2_/Fe(111) slab as a model, because the hexagonal unit cell on the Fe(111) surface structurally matches the surface cell of MoS_2_ and a better lattice match is achieved between both materials. The size of the unit cell of the $\sqrt{3}\times \sqrt{3}$ MoS_2_ slab is 5.473 × 5.473 Å with an angle of 120°, which is a good fit with the Fe(111) surface with the dimensions of 4.009 × 4.009 Å and an angle of 120°. In our setup, the MoS_2_ was subjected to a small strain (≈2.4%) to make it commensurable with Fe(111), and the effect of the lattice mismatch on the electronic structure of the MoS_2_ was negligible.

After optimizing the structures from four initial configurations in an interface with the monolayer MoS_2_, i.e., the top, bridge, fcc hollow, and hcp hollow sites formed by the three Fe atoms in the top layer, we obtained the most stable configurations of the MoS_2_/Fe(111) interfaces, as shown in Figure 1c,d. The S atom in the dotted red circle was located at the bridge site of two Fe atoms. In terms of the binding energy per interfacial sulfur atom, calculated as

(3) $$E_{b}=(E_{{\mathrm{MoS}}_{2}/\mathrm{Fe}}-E_{\mathrm{Fe}}-E_{{\mathrm{MoS}}_{2}})/N_{s}$$

where N_S_ is the number of interfacial sulfur atoms and N_S_ = 27 for the calculated model, the Fe(111) surface had a medium adhesion with MoS_2_ with an *E*_b_ = −0.41 eV, which is larger than the weak interaction of MoS_2_–Au but smaller than the strong interaction of MoS_2_–Sc or MoS_2_–Ti [28]. The planar plane of the Fe(111) surface was distorted since the top layer Fe atoms were stretched by the MoS_2_. The average distance between the interfacial sulfur atoms with the top layer Fe atoms was 1.901 Å. The short interfacial distance also suggests that MoS_2_ forms a stable coating on an iron surface, which is beneficial for preventing hydrogen from transiting to Fe.

It is interesting to study the potential energy of the H atom adsorbing on the MoS_2_/Fe surface, and then moving through the interface region from the MoS_2_ part to the Fe substrate. To find the most energetically stable H adsorption site on the MoS_2_/Fe surface, we examined five possible initial positions for H on the clean monolayer MoS_2_, i.e., the top and bridge sites of Mo atoms and the top, bridge, and hollow sites of S atoms. In terms of the adsorption energy, we obtained the most energetically stable configuration, that is, H initially locates at the Mo bridge site and then binds to the surface S atom after structural relaxation. In this configuration, the atomic H chemically binds to the top surface of S with an *E*_ads_ of 1.64 eV and a S–H distance of 1.417 Å (Table 1), in agreement with previous reports [29]. Figure 1c,d depicts the side and top views of the most stable H adsorption geometries on the MoS_2_/Fe surface, a similar adsorption configuration is obtained with an *E*_ads_ of 1.26 eV. The positive *E*_ads_ suggests that the H_2_ molecule cannot spontaneously dissociate on the MoS_2_/Fe film, which effectively suppresses the H dissociative adsorption. Experimental measurements have also demonstrated that the planar surface monolayer MoS_2_ is chemically rather inert while the edge sites of clusters and the defected layers are chemically reactive [30,31], which is consistent with the present results. Furthermore, as an atomic H migrates into the interface, it has been reported that an energy barrier of about 0.57 eV is required to pass through the center of the hexagonal structure in MoS_2_ [29], because there is a repulsive force induced by a strong electron cloud of MoS_2_. This energy barrier is up to 6.56 eV for the H_2_ molecule passing through the hole of the monolayer MoS_2_ [29], indicating that H is difficult to diffuse into the other side of MoS_2_. Even if H successfully moves to the interface at a high temperature or under a large tensile strain, the energy for binding to the Fe-contacted S atom is 0.15 eV higher than that of the up-surface S atom. All the results demonstrate that a MoS_2_ coating acts as an energy barrier which interrupts hydrogen penetration by the formation of S–H bonds.

**Figure 3:**
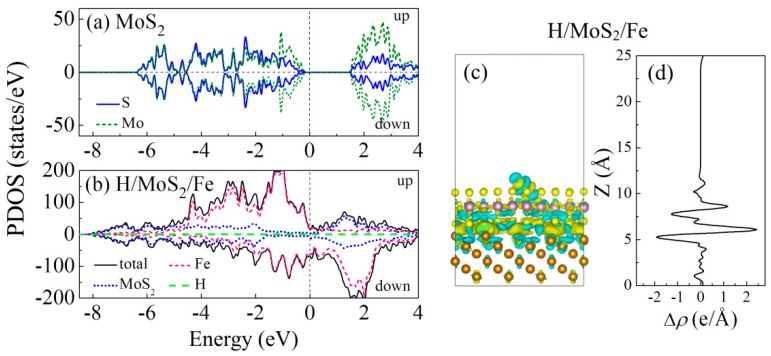
(**a**) Projected density of states on S and Mo atoms in a clean monolayer MoS_2_. (**b**) Total density of states (DOS) and PDOS on Fe, MoS_2_, and H in the H/MoS_2_/Fe system. (**c**) Charge transfer density between the H, the MoS_2_, and the Fe(111) film. The yellow and blue colors represent electron accumulation and depletion, respectively. The charge density isosurface was set to 0.003 e Å^−3^. (**d**) Interfacial charge transfer between the H, the MoS_2_, and the Fe(111) film as a function of the z coordinate perpendicular to the surface.

To gain insight into the H prevention effect of the MoS_2_ coating, Figure 3a,b depicts the PDOS of the clean MoS_2_ film and the H-adsorbed MoS_2_/Fe(111) film. As seen from Figure 3a, a perfect monolayer MoS_2_ is a semiconductor with a band gap of 1.75 eV, in accordance with literature results [32]. There is no net magnetic moment for S and Mo atoms. When it is fabricated on Fe(111) film, the gap of MoS_2_ significantly shifts down in energy, as displayed in Figure 3b, i.e., the upward shift of the Fermi level, indicating that the MoS_2_ is n-doped on the Fe(111) surface. Furthermore, the electronic states of S and Mo are broadened within the band gap (−1.6~0.1 eV), due to the strong orbital interfacial hybridization between the Fe and S atoms. The H/MoS_2_/Fe(111) film still presents a ferromagnetic property, with split up and down orbitals for the PDOS in Figure 3b. This magnetic behavior mainly stems from the single *d* electron provided by the Fe atom. There is also a weak magnetic moment on the MoS_2_ layer induced by the Fe contact, in which Mo reaches 0.05 μ_B_ and the interface S atoms are less than 0.02 μ_B_, in agreement with previous calculations for MoS_2_ on a single layer of Fe [33]. Moreover, for the H-adsorbed MoS_2_/Fe(111) film, the total DOS at the Fermi level, *E*_F_, is mainly contributed from the down spin of the Fe 3*d* states.

Figure 3c,d plots the interfacial charge transfer of the H-adsorbed MoS_2_/Fe(111) film. The differential charge density at the interface is defined as

(4) $$\Delta \rho =\rho_{H/{\mathrm{MoS}}_{2}/\mathrm{Fe}}-(\rho_{{\mathrm{MoS}}_{2}}+\rho_{\mathrm{Fe}}+\rho_{H})$$

where $\rho_{H/{\mathrm{MoS}}_{2}/\mathrm{Fe}}$, $\rho_{{\mathrm{MoS}}_{2}}$, $\rho_{\mathrm{Fe}}$, and $\rho_{H}$ are the charge densities of the H/MoS_2_/Fe, MoS_2_, and Fe(111) films and the H atom. Firstly, at the MoS_2_–Fe interface, there is a large amount of charge transfer from the top two layers of Fe to the interfacial S atoms in MoS_2_, a total of 4.91 e (calculated by Bader charge analysis). A strong binding between MoS_2_ and Fe with a distance of 1.901 Å allows a strong wave-function overlap between the Fe and the S states. Secondly, the adsorbed H donates 0.018 e to the neighboring S atom by forming a S–H bond, as listed in Table 1.

The above results demonstrate that the MoS_2_ coating on the Fe(111) film effectively prevents H adsorption on the iron surface or permeation into the bulk. The influence of the MoS_2_ coating on corrosion resistance is also reflected by work function, which is a sensitive parameter for the corrosive behavior of materials. Previous studies have suggested that materials with a lower work function possess a lower corrosion potential and consequently become easily corroded [34,35]. The work function (W_F_) is calculated as the difference between the vacuum level, *E*_vacuum_, and the Fermi energy, *E*_F_:

(5) $$W_{F}=E_{\mathrm{vacuum}}-E_{F}$$

**Figure 4:**
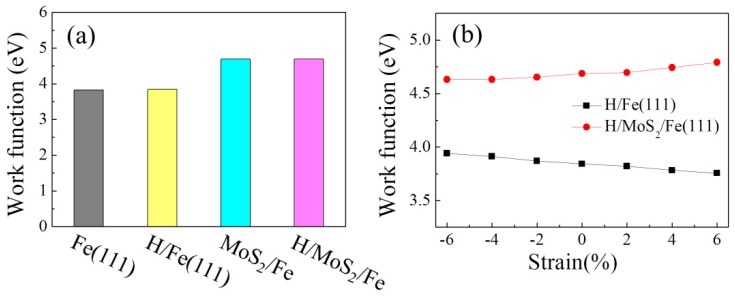
(**a**) The work function of the Fe(111) films and the MoS_2_/Fe(111) films without and with H adsorption. (**b**) The work function of the H-adsorbed Fe(111) films and the H-adsorbed MoS_2_/Fe(111) films with a strain of up to ±6%.

Here, W_F_ reflects the electronic energy level, so it is related to its electrostatic potential. The work function of a clean Fe(111) surface is 3.823 eV, which is lower than the closely packed crystallographic plane (low-index) Fe(110) surface [36]. After H adsorption, the W_F_ is slightly enhanced to 3.842 eV, as listed in Table 1, because the H atom withdraws electrons from the Fe and a weak dipole pointing inward (from H to Fe) is formed. Figure 4a plots the W_F_ of the H-adsorbed Fe(111) with and without the MoS_2_ coating. The W_F_ of the H/MoS_2_/Fe(111) system significantly increases to 4.688 eV, which is lifted by 0.846 eV compared to that without the MoS_2_ coating, since the H-adsorbed MoS_2_ exhibits a high W_F_ of 4.705 eV. It is indispensable to understand why the coating and H adsorption change the work function of these films.

There could be different factors, such as epitaxial strain, structural deformation, hydrogen, and the external environment, affecting the mechanical properties of steels. Experimental studies have suggested that stress corrosion cracking and hydrogen embrittlement are the most dominating damages for steels [17,18]. Next, we discuss the work function changes associated with the H adsorption in the strained system. A biaxial strain by rescaling the in-plane lattice constant was applied to the H/Fe(111) and the H/MoS_2_/Fe films. Without the MoS_2_ coating, the W_F_ of the H/Fe(111) system increases/decreases by ~0.1 eV under compressive/tensile strain up to 6%, as plotted in Figure 4b. For instance, the W_F_ is 3.939 eV under 6% compressive strain and 3.756 eV under 6% tensile strain. This property is consistent with the experimental and theoretical results that the W_F_ of metals decreases with tensile strain [37,38]. In the case of the H/MoS_2_/Fe(111) system, the W_F_ shows an opposite response to strain, i.e., it decreases to 4.631 eV under 6% compressive strain while it increases to 4.791 eV under 6% tensile strain. The changes in the W_F_ of the MoS_2_-coated film can be attributed to the strained MoS_2_, which exhibited a W_F_ decrease under compressive strain and a W_F_ increase under tensile strain [39,40]. Under both compressive and tensile strains, a much higher work function is observed for the H/MoS_2_/Fe film than that of the system without the MoS_2_ coating, indicating that the strained Fe(111) film with the MoS_2_ coating becomes more corrosion resistant.

**Figure 5:**
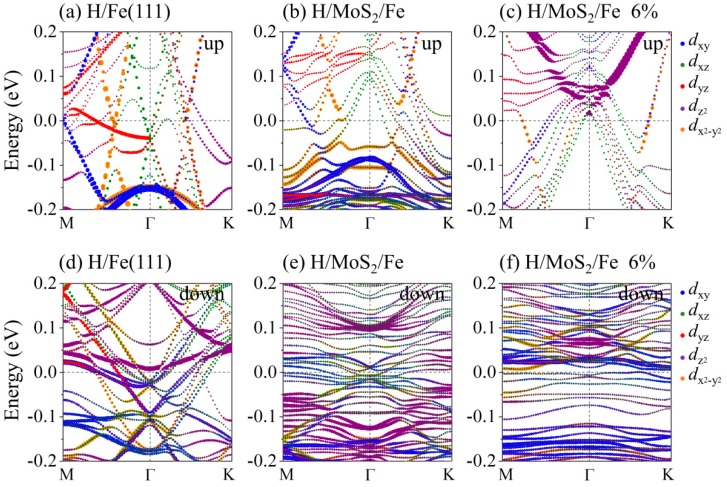
Band structures along the M-Γ-K directions of (**a**,**d**) the H-adsorbed Fe(111), (**b**,**e**) the H-adsorbed MoS_2_/Fe film, and (**c**,**f**) the H-adsorbed MoS_2_/Fe film with 6% tensile strain for up and down spins. Blue, green, red, purple, and orange lines on the bands illustrate the contribution from *d*_xy_, *d*_xz_, *d*_yz_, $d_{z^{2}}$, and $d_{x^{2}-y^{2}}$ states.

The effect of the coating and the strain on the work function depends on how they affect the Fermi energy. To clarify the origin of the W_F_ changes in the strained systems, Figure 5 plots the orbital-resolved band structures for the H-adsorbed Fe(111) film without or with the MoS_2_ coating. Figure 5a–c shows the band structures for spin-up orbitals, corresponding with Figure 5d–f for the spin-down orbitals. Only five *d* orbitals are presented in Figure 5, because the energy bands near the Fermi level are mainly contributed from Fe 3*d* orbitals, as indicated by the PDOS in Figure 3b. Comparing the H/Fe(111) (Figure 5a,d) with the H/MoS_2_/Fe system (Figure 5b,e), one prominent property is that the bands near the Fermi level become less dispersive, especially for the down spin in Figure 5e. The flatter bands lead to a low Fermi velocity, indicating a quite low Fermi energy, and then give rise to an enhancement in the work function of H/MoS_2_/Fe. As a 6% tensile strain is applied to the H/MoS_2_/Fe(111) film, the flatter feature at the Fermi level is more pronounced in Figure 5f, suggesting that the W_F_ of the H/MoS_2_/Fe system increases with tensile strain. The increased W_F_ indicates that the MoS_2_-coated surface becomes more corrosion resistant.

## 4. Conclusions

In conclusion, we studied the MoS_2_ coating on an iron surface as a protective barrier against H damage. The monolayer MoS_2_ can be stably coated on the Fe(111) surface with a binding energy of −0.41 eV per surface S atom and an interfacial distance of 1.901 Å. Through the characterization of hydrogen-adsorbed MoS_2_/Fe(111), it was identified that MoS_2_ can effectively prevent hydrogen adsorption and penetration by the formation of a S–H bond. The hydrogen adsorption energy on the Fe(111) surface is enhanced from −0.55 eV to 1.26 eV with the MoS_2_ coating, suggesting that monolayer MoS_2_ can effectively inhibit the dissociative adsorption of hydrogen molecules. In addition, the work function of MoS_2_-coated Fe(111) films substantially increases by 0.846 eV, further indicating a more corrosion resistant property of the MoS_2_-coated Fe(111) films owing to their improved surface properties. The results demonstrate that the MoS_2_ coating is a proper barrier for H adsorption or permeation and can effectively avoid hydrogen damage. Based on the protective performance of monolayer MoS_2_, multiple layers of MoS_2_ or a thin film are expected to possess a better hydrogen prevention effect due to more barriers for hydrogen diffusion. Since it is still a challenge to produce a high quality monolayer MoS_2_ on a large scale on steels, we suggest that coating with multiple layers of MoS_2_ film might be more applicable.

## Figures and Tables

**Table 1:** The adsorption energy (*E*_ads_), the closest H–Fe distance (*d*_H-Fe_) or H–S distance (*d*_H-S_), the amount of charge transfer of the adsorbed H atom (ΔQ_H_), and the work function (W_F_) of H adsorptions on the Fe(111), MoS_2_, and MoS_2_/Fe(111) films. *E*_ads_ is calculated by Equation (1).

	*E*_ads_ (eV)	*d*_H-Fe_/*d*_H-S_ (Å)	ΔQ_H_ (e)	W_F_ (eV)
H on Fe(111)	−0.55	1.636	0.355	3.842
H on MoS_2_	1.64	1.417	−0.042	4.705
H on MoS_2_/Fe(111)	1.26	1.425	−0.018	4.688
